# Haoya Wang Et Al.: Circadian Rhythm Disruption Promotes Tumor Progression Through Upregulated Glycolysis

**DOI:** 10.1002/cam4.71138

**Published:** 2025-08-07

**Authors:** Haoya Wang, Di Wu, Jie Li, Binghe Zhao, Lu Liu, Xinxin Wang

**Affiliations:** ^1^ The First Medical Center of PLA General Hospital Beijing China

**Keywords:** aerobic glycolysis (Warburg effect), chronotherapy, circadian rhythm, glycolytic inhibitors, tumor microenvironment

## Abstract

**Background:**

Aberrant activation of glycolysis (Warburg effect) constitutes a key metabolic reprogramming feature in malignant tumors, serving as a critical mechanism facilitating tumor development. Within the tumor microenvironment, this glycolytic reprogramming emerges in diverse cellular components, including cancer cells, immune cells (e.g., myeloid‐derived suppressor cells and tumor‐associated macrophages), and fibroblasts, thereby establishing a microenvironment that promotes tumor invasion and metastasis. Recent studies have revealed that the endogenous circadian system orchestrates glycolysis processes through multiple pathways, where circadian rhythm disruption frequently manifests as upregulated glycolysis with pro‐tumorigenic consequences.

**Methods:**

This review summarizes the specific mechanisms through which circadian rhythm disruption regulates the reprogramming of glycolytic metabolism in the tumor microenvironment. Emerging chronotherapeutic strategies focus on targeting glycolytic pathways.

**Conclusions:**

The reprogramming promotes enhanced glycolysis, ultimately accelerating tumor progression. Combination therapy with glycolysis inhibitors has the potential to further improve efficacy when optimized for time. Future research should prioritize unraveling the complex interplay between circadian rhythms, glycolysis, and the tumor microenvironment to advance more effective therapeutic interventions.

**Abbreviations**
2‐DG: 2‐deoxy‐D‐glucose
*BMAL1*
: brain and muscle ARNT‐Like 1
CAFs: cancer‐associated fibroblasts
*CLOCK*
: circadian locomotor output cycles kaput
CRC: colorectal cancer
*CRY*
: cryptochrome
CSCs: cancer stem cells
DCA: dichloroacetate
EMT: epithelial‐mesenchymal transition
*F. nucleatum*
: *Fusobacterium nucleatum*
GLP‐1: glucagon‐like peptide 1
GLUTs: glucose transporters
GSC: glioma stem cell
GSK‐3β: glycogen synthase kinase 3β
HIF‐1α: hypoxia‐inducible factor‐1α
HKs: hexokinases
ICB: immune checkpoint blockade
LDHA: lactate dehydrogenase
MCTs: monocarboxylate transporters
MDSCs: myeloid‐derived suppressor cells
NAD+: nicotinamide adenine dinucleotide
NFs: normal fibroblasts
*NPAS2*
: neuronal PAS domain protein 2
OSCC: oral squamous cell carcinoma
OXPHOS: oxidative phosphorylation
PDK: pyruvate dehydrogenase kinase
*PER*
: period
PFKFB3: 6‐phosphofructo‐2‐kinase/fructose‐2,6‐bisphosphatase 3
PKM2: pyruvate kinase M2
SCFAs: short‐chain fatty acids
SCN: suprachiasmatic nucleus
TAMs: tumor‐associated macrophages
TCA: taurocholic acid
TCA cycle: tricarboxylic acid cycle
TGF‐β: transforming growth factor‐β
TTFLs: transcriptional‐translational feedback loops

## 1 Introduction

**FIGURE 1:**
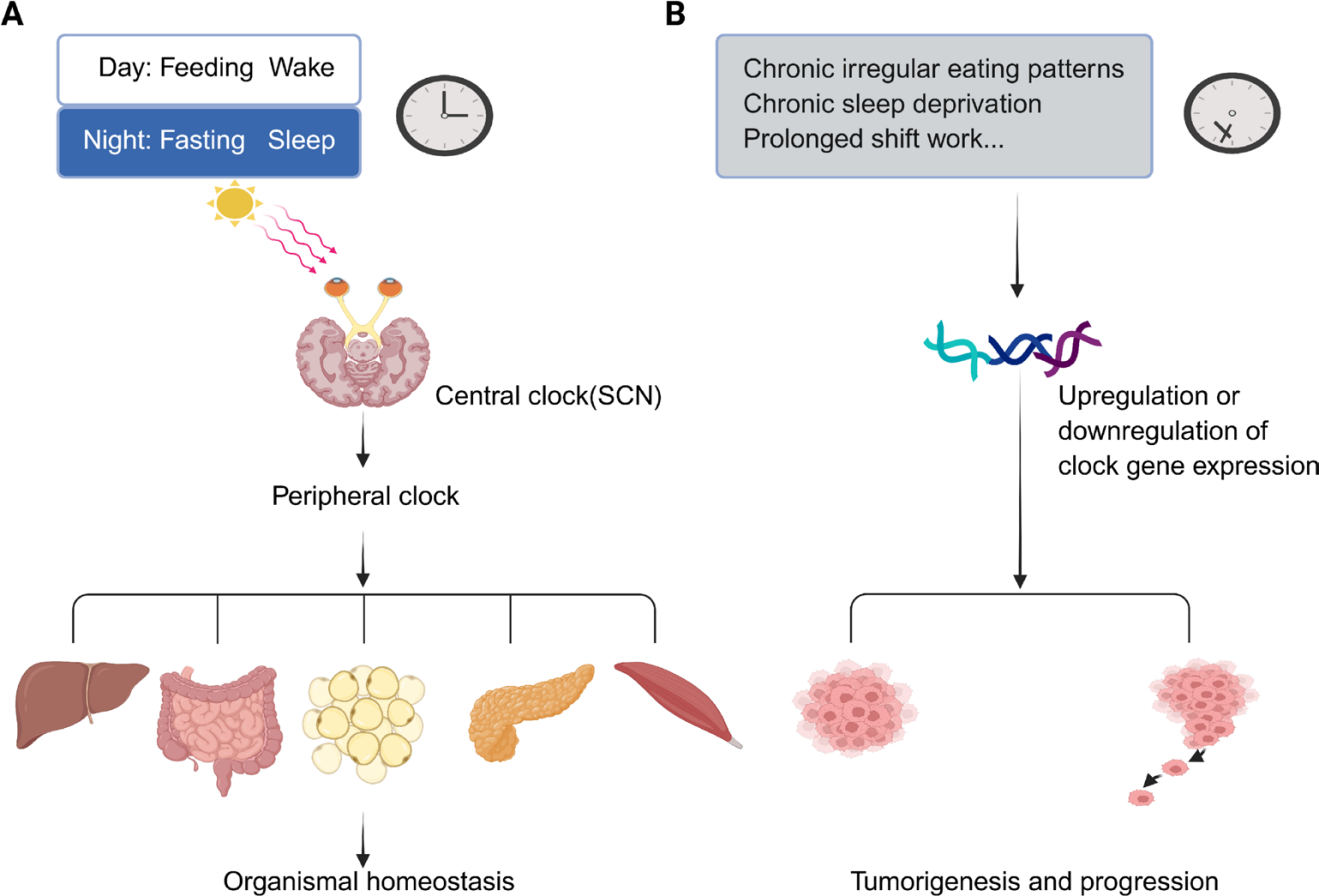
Relationship between circadian rhythms and homeostasis. (A) Physiological circadian rhythms coordinating sleep–wake cycles and feeding–fasting cycles collaboratively maintain organismal homeostasis through precise regulation of the central clock (suprachiasmatic nucleus, SCN) and subsequent synchronization of peripheral clocks in organs and tissues, including hepatic, gastrointestinal, adipose, pancreatic, and muscle. (B) Circadian disruption arising from chronic irregular eating patterns, chronic sleep deprivation, or prolonged shift work induces transcriptional dysregulation of core clock components. This molecular perturbation subsequently leads to homeostatic imbalance, creating a permissive microenvironment conducive to both tumorigenesis and progression.

The circadian clock, also known as the biological clock, is primarily governed by core clock genes including *BMAL1* (Brain and Muscle ARNT‐Like 1) and *CLOCK* (Circadian Locomotor Output Cycles Kaput), which establish 24‐h oscillations to maintain organismal homeostasis [1]. Mammalian circadian regulation involves a hierarchical organization in that the central clock in the hypothalamic suprachiasmatic nucleus (SCN) synchronizes with peripheral clocks distributed across tissues, coordinating cellular activities with environmental cues [2, 3]. The SCN integrates light signals detected by retinal melanopsin (a G‐protein‐coupled receptor) and relays this information to peripheral systems, thereby entraining endogenous rhythms like the sleep–wake cycle [4, 5]. In addition, peripheral clocks exhibit both SCN‐independent functionality and the capacity to synchronize the central clock through non‐photic signals [6] (Figure 1A). This circadian network regulates diverse physiological systems, including the liver, pancreas, muscles, adipose tissue, and even mitochondria [7, 8]. At the molecular level, the circadian machinery operates through transcriptional‐translational feedback loops (TTFLs) involving cryptochrome (*CRY*), period (*PER*) genes, ROR nuclear receptors, and REV‐ERB transcription factors. These components interact with the BMAL1‐CLOCK heterodimer to generate self‐sustained oscillations through reciprocal regulatory loops [9, 10]. Chronic disruption of circadian rhythms (e.g., sleep–wake cycle and feeding–fasting cycle disturbances) is increasingly recognized as a contributor to pathological states including chronic inflammation, metabolic dysregulation, and carcinogenesis [11, 12, 13]. Growing evidence highlights the mechanistic links between circadian misalignment and tumor biology, prompting an intensified investigation into their pathophysiological interactions [13].

The glycolytic pathway serves as a fundamental energy‐producing mechanism shared by both anaerobic and aerobic cellular processes, constituting the primary metabolic route in human tissues. Under physiological conditions, glycolytic pathway end‐product pyruvate enters mitochondria for oxidative phosphorylation, whereas under hypoxia, cytoplasmic pyruvate is reduced to lactate via lactate dehydrogenase to complete glycolysis [14]. Metabolic reprogramming, a recognized hallmark of malignancy [15], extends beyond cancer cells to reshape the tumor microenvironment through enhanced glycolytic activity in stromal and immune cells [16, 17, 18]. Elevated glycolysis in tumor cells correlates strongly with cancer progression across multiple malignancies [19, 20, 21]. In normal physiology, circadian regulation maintains rhythmic oscillations of glycolytic activity, evidenced by preserved glycolytic rhythms even in enucleated human erythrocytes [22]. Tumor cells, however, exhibit dysregulated cellular metabolism with aberrant glycolytic flux that fuels proliferation and metastasis [23]. From the perspective of glycolysis, this metabolic reprogramming is characterized by enhanced glucose uptake and increased lactate production [24]. The resultant acidic, lactate‐rich microenvironment exerts significant influence on the tumor microenvironment by facilitating angiogenesis, epithelial‐mesenchymal transition, extracellular matrix remodeling, and immune evasion mechanisms [25], thereby collectively contributing to tumor progression. Emerging research suggests that circadian disruption might also influence the glycolysis of other cells in the tumor microenvironment, providing an invasive and metastatic environment for malignancies [26, 27]. These findings underpin chronotherapeutic strategies targeting glycolysis, with potential synergy when combined with conventional treatments and emerging modalities, offering novel approaches for cancer management [10].

## 2 Enhanced Glycolysis Drives Metabolic Reprogramming Within the Tumor Microenvironment

Glycolysis serves as a core energy‐producing pathway in cellular metabolism [28], rapidly generating ATP for biological processes while supplying precursors for biosynthetic reactions. In normoxic conditions, pyruvate in normal cells is transported into mitochondria, where it undergoes oxidative phosphorylation via the electron transport chain to produce substantial ATP for cellular energy demands [29]. By contrast, even under sufficient oxygen conditions, tumor cells preferentially convert cytoplasmic pyruvate into lactate through aerobic glycolysis for energy production, a metabolic adaptation first described by Warburg in the 1920s and later named the Warburg effect [30]. This metabolic shift from oxidative phosphorylation to aerobic glycolysis represents a hallmark feature of malignant progression, critically contributing to tumor proliferation and invasive potential [31]. Such glycolytic reprogramming is consistently observed across major cellular components of the tumor microenvironment, including cancer cells, immune cells, and stromal fibroblasts.

### 2.1 Cancer Cells

In cancer cells, elevated glycolytic flux correlates with upregulated expression of glucose transporters (GLUTs) and hexokinases (HKs) [32]. GLUTs constitute a transmembrane protein family comprising 14 isoforms (GLUT1‐GLUT14) that facilitate cellular glucose uptake, whereas HKs represent rate‐limiting glycolytic enzymes encoded by distinct genes and categorized into four isoforms (HK1‐HK4, with HK4 designated as glucokinase). They exhibit tissue‐specific distribution patterns [33]. Cancer cell lines demonstrate isoform‐specific GLUT/HK expression profiles. For instance, breast cancer cells exhibit characteristic upregulation of GLUT1 and GLUT3 alongside malignant transformation, with recent studies identifying GLUT12 as a novel therapeutic target for glucose transport modulation [34]. HK2, overexpressed in numerous malignancies, has emerged as a prominent therapeutic target because of its association with tumor proliferation, metastatic dissemination, and resistance to radiotherapy and chemotherapy [35]. Under hypoxic conditions, cancer stem cells (CSCs) preferentially utilize aerobic glycolysis to sustain self‐renewal capacity and invasive properties [36]. HK2 overexpression in small‐cell lung cancer stem cells enhances intracellular aerobic glycolysis to preserve stem‐like characteristics [37].

### 2.2 Immune Cells

The acidic microenvironment generated by these tumor cell metabolic alterations contributes to the establishment of an immunosuppressive tumor microenvironment [38]. As critical components of the tumor microenvironment, most immune cells undergo metabolic remodeling that drives glycolytic dependency for energy production [39]. Tumor cells actively compete with antitumor immune effectors (including CD8+ T cells, NK cells, and dendritic cells) for glucose utilization, while concurrently promoting glycolysis in immunosuppressive populations such as myeloid‐derived suppressor cells (MDSCs) [40]. Tumor‐associated MDSCs in breast cancer mouse models indicated upregulation of nearly all glycolysis‐related enzymes and enhanced glucose uptake, whereas administration of glycolytic inhibitors reduced MDSC proportions and suppressed tumor growth [41]. Macrophages exist in two polarized states: pro‐inflammatory M1 and anti‐inflammatory M2 phenotypes [42]. Although tumor‐associated macrophages (TAMs) typically express M2‐like polarization, their metabolic profile resembles M1‐type macrophages through elevated glycolytic flux [43]. This metabolic preference drives an immunosuppressive functional phenotype, impairing CD8+ T cell effector functions and facilitating immune evasion.

### 2.3 Fibroblasts

Cancer‐associated fibroblasts (CAFs), as major cellular constituents within the tumor stroma, facilitate tumor progression through extracellular matrix remodeling and immunosuppression [44]. These activated fibroblasts are subjected to tumor‐driven metabolic reprogramming toward aerobic glycolysis [45]. Comparative analysis of CAFs from pancreatic cancer patients versus normal fibroblasts (NFs) from pancreatic injury patients revealed upregulated expression of glycolytic enzymes in CAFs, including lactate dehydrogenase(LDHA) and pyruvate kinase M2(PKM2), accompanied by significantly enhanced glycolytic activity [46]. Within the tumor microenvironment, some cytokines and transcription factors, such as TGF‐β and HIF‐1α, not only induce fibroblast‐to‐CAF transformation but also perpetuate their glycolytic phenotype [47, 48]. Furthermore, bidirectional metabolic crosstalk occurs between cancer cells and CAFs. Through this interaction, cancer cell‐secreted hydrogen peroxide induces oxidative stress and promotes aerobic glycolysis in CAFs, producing metabolic substrates (e.g., pyruvate and lactate) that are subsequently shuttled to cancer cells for ATP generation via oxidative phosphorylation—a phenomenon termed the “reverse Warburg effect”. This metabolic coupling ultimately enhances cancer cell proliferation, invasion, and metastasis [49, 50].

## 3 Circadian Rhythm Disruption Promotes Tumor Progression via Multiple Mechanisms Involving Upregulated Glycolysis

**FIGURE 2:**
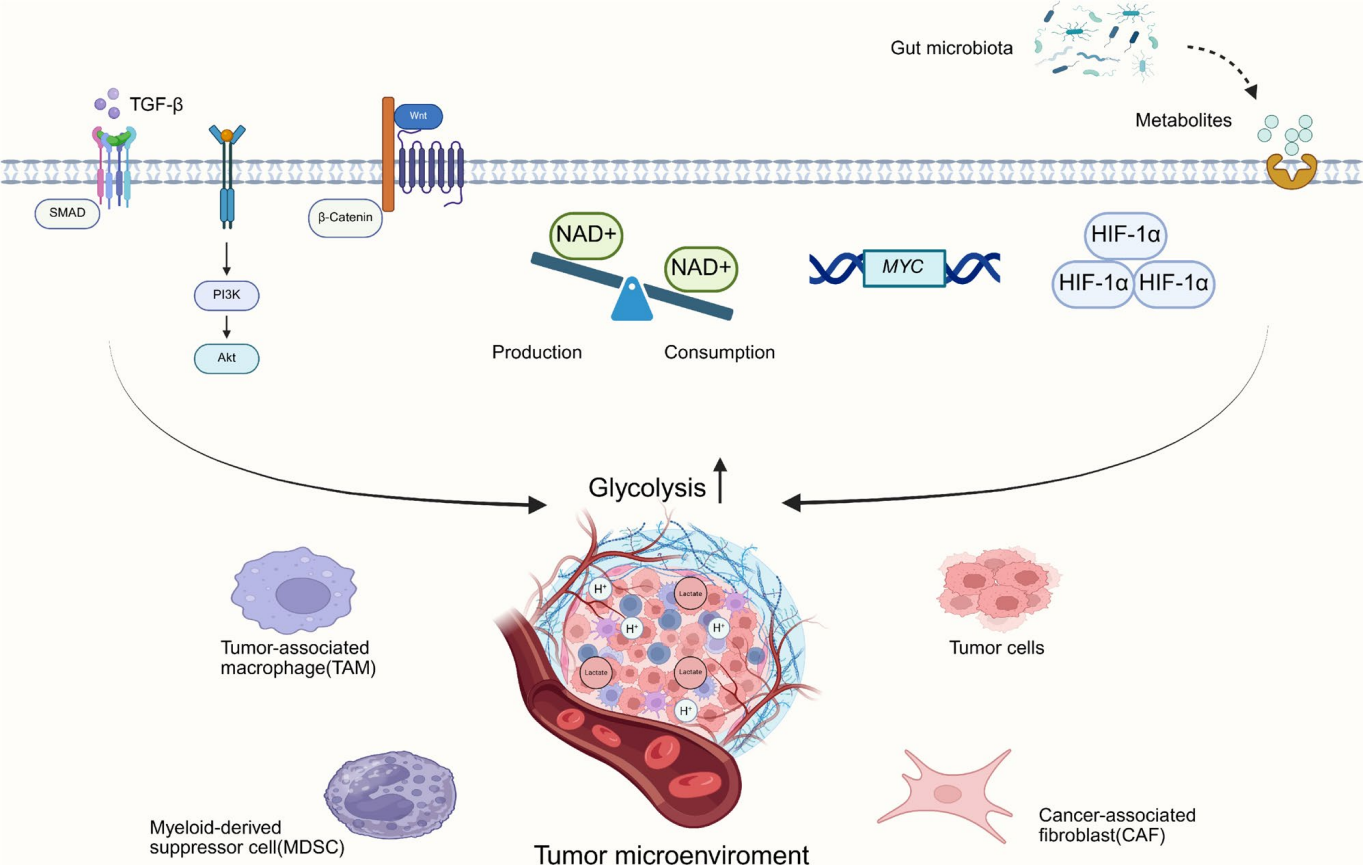
Mechanisms linking circadian rhythm disruption to tumor progression through glycolytic reprogramming in the tumor microenvironment. Chronodisruption drives microenvironmental glycolytic upregulation via: (1) related signaling molecules (WNT/β‐catenin, PI3K‐Akt, TGF‐β/Smad, and NAD+), (2) genetic and transcriptional regulation (e.g., *MYC* and HIF‐1α), and (3) gut microbiota and associated metabolites.

A study by Susanne E. la Fleur et al. manifested circadian‐dependent glucose regulation in SCN‐intact rats, with plasma glucose concentrations and tissue glucose uptake troughing at the light phase onset and peaking during the dark phase. SCN‐disrupted rats exhibited elevated glucose uptake equivalent to dark phase levels in controls, accompanied by rhythm loss [51]. These findings indicate central circadian control of glucose metabolism. In malignant cells, such circadian regulation is frequently perturbed, leading to dysregulation of core metabolic pathways, including glycolytic flux [52, 53]. Mechanistically, circadian disruption activates oncogenic glycolysis (Figure 2) that potentiates tumor growth and metastasis. High glycolysis is a metabolic hallmark of tumor progression [54]. Multiple cell populations within the tumor microenvironment similarly upregulate glycolysis upon circadian disruption (Figure 2), fostering a microenvironment that promotes tumor proliferation and invasion [55]. Furthermore, studies indicate that altered clock gene expression occurs more frequently than mutations during carcinogenesis [56], where overexpression or loss of specific clock genes correlates with tumor progression (Figure 1B). Circadian rhythms, glycolytic metabolism, and tumorigenesis exhibit dynamic interplay, with clock gene dysregulation (overexpression or suppression) directly linked to enhanced cancer cell proliferation [57].

### 3.1 Circadian Rhythm Disruption Promotes Tumor Progression Through Related Signaling Molecules' Regulation of Glycolysis

The canonical WNT/β‐catenin signaling pathway regulates cellular metabolism, differentiation, and epithelial‐mesenchymal transition (EMT) [58, 59]. It is essential for the cell cycle, immune responses, and circadian rhythms [60]. When dysregulated, this pathway demonstrates activation in most malignancies, driving aerobic glycolysis to sustain CSC stemness [61]. Clock gene changes across malignancies often coexist with this oncogenic signaling [62]. *Bmal1* overexpression maintains glioma stem cell (GSC) stemness via WNT pathway activation, conferring therapeutic resistance and relapse risk [63, 64]. The PI3K/AKT signaling pathway, among the most frequently dysregulated pathways in cancer, inhibits glycogen synthase kinase 3β (GSK‐3β) to suppress glycogen synthesis while activating key glycolytic enzymes, promoting glycolysis, and thereby regulating cellular proliferation [65]. *PER2*‐silenced oral squamous cell carcinoma (OSCC) cells exhibited upregulated PI3K and phospho‐Akt(p‐Akt) expression, accompanied by enhanced activity of key glycolytic enzymes (HK2, PKM2, and LDHA), with resultant elevated glycolytic flux driving proliferation and suppressing apoptosis [66]. Within the tumor microenvironment, transforming growth factor‐β(TGF‐β) secretion by stromal constituents facilitates malignant invasion, metastasis, and treatment refractoriness [67]. Beyond CAF activation, TGF‐β critically modulates fibroblast metabolism via TGF‐β1/Smad signaling, which under hypoxic conditions synergistically induces MCT4/glycolytic enzyme overexpression (LDHA, PKM2, and HK2) and tumor EMT to potentiate metastasis [47, 68]. The crosstalk between the circadian clock and TGF‐β is mediated by multiple molecular mechanisms. The circadian clock regulates TGF‐β signaling through both direct and indirect pathways [27, 69]. *Bmal1* deficiency elevates fibrinolysis protease activity, triggering TGF‐β‐mediated expansion of α‐SMA+ myoCAFs that accelerate colorectal cancer (CRC) progression [27].

Nicotinamide adenine dinucleotide (NAD+), an essential cofactor for dehydrogenases, mediates electron transfer during mitochondrial oxidative phosphorylation. Cellular NAD+ depletion disrupts mitochondrial function to supply carbon sources for fatty acid synthesis, while simultaneously stimulating NAD+ regeneration from NADH, thereby enhancing pyruvate‐to‐lactate conversion. These metabolic shifts collectively drive glycolytic dominance under NAD+ ‐deficient conditions. Sirtuins are a family of NAD+ ‐dependent lysine deacetylases. Their activity depends on the concentration of NAD+. Under NAD+ ‐deficient conditions, compromised deacetylase activity triggers mitochondrial enzyme acetylation, thereby augmenting glycolytic flux. In cancer cells, *CLOCK* gene disruption induces NAD+ imbalance through consumption outpacing production, which sustains biosynthetic requirements while driving NAD+ depletion‐mediated aerobic glycolysis and tumor proliferation [70, 71]. These coordinated signaling molecules show that circadian dysregulation governs tumor glycolytic reprogramming, revealing novel therapeutic targets.

### 3.2 Circadian Rhythm Disruption Promotes Tumor Progression Through Genetic and Transcriptional Regulation of Glycolysis

Proto‐oncogene activation and tumor suppressor gene inactivation represent fundamental oncogenic mechanisms [72]. Some proto‐oncogenes and tumor suppressor genes, including *MYC* and *TP53*, are circadian‐regulated and categorized as Clock Controlled Genes (CCGs) [73]. As a transcription factor, MYC manifests reciprocal regulatory effects on the circadian clock. Aberrantly activated *MYC* promotes the expression of the circadian repressor REV‐ERB, which subsequently suppresses *BMAL1* mRNA transcription. Importantly, *MYC* activation in human osteosarcoma cells was first shown to enhance glucose flux and lactate production [74]. *C‐MYC* orchestrates metabolic adaptations in cancer cells by upregulating GLUT1 and nearly all glycolytic enzymes [75]. *Per2*‐deficient lung tumor cells exhibit an elevated *c‐Myc* expression accompanied by accelerated glucose consumption, augmented lactate output, and enhanced mitochondrial Tricarboxylic Acid Cycle (TCA cycle) activity [76]. Furthermore, *Bmal1* knockdown cooperates with heterozygous *Apc* deletion to induce WNT pathway hyperactivation, which upregulates *c‐Myc* expression and augments glycolytic metabolism, ultimately promoting colorectal cancer cell proliferation [77].

Hypoxia‐inducible factor‐1α (HIF‐1α), a key transcriptional regulator, becomes activated in hypoxic tumor microenvironments [78]. Under such conditions, it can coordinate multiple downstream targets to promote tumor invasiveness and therapy resistance [18]. As a central modulator of glycolysis, HIF‐1α enhances glycolytic flux through the upregulation of glucose transporters and enzymes involved in glycolysis, while concurrently suppressing oxidative phosphorylation (OXPHOS) [79]. In addition, HIF‐1α exhibits rhythmic fluctuations in transcriptional activity and can reciprocally influence circadian rhythm regulation [80]. Circadian rhythm disruption‐mediated HIF‐1α overexpression drives tumor progression. In myeloid‐specific *Bmal1* knockout TAMs, elevated HIF‐1α expression promotes aerobic glycolysis upregulation. That reduces CD8+ T cell infiltration in the tumor microenvironment, fostering an immunosuppressive microenvironment that correlates with increased tumor burden in melanoma‐bearing mice [26]. HIF‐1α serves as a direct transcriptional target of the circadian regulator neuronal PAS domain protein 2 (*NPAS2*). Hepatocellular carcinoma cells with *NPAS2* overexpression exhibit increased *HIF‐1α* mRNA levels, upregulated glycolytic genes (including *GLUT1* and *HK2*), and elevated glucose uptake accompanied by excessive lactate production, collectively promoting cancer cell invasion and metastasis [81]. These findings indicate that circadian rhythm disruption potently enhances tumor glycolytic metabolism through transcriptional reprogramming, thereby accelerating malignant progression.

### 3.3 Circadian Rhythm Disruption Promotes Tumor Progression Through Gut Microbiota and Metabolites' Regulation of Glycolysis

The gut microbiota represents the indigenous microbial ecosystem that synthesizes amino acids, vitamins, and essential small molecules for host development. These microbial communities regulate macronutrient metabolism (carbohydrates, proteins, and lipids) and enhance micronutrient absorption. Predominant phyla comprise Firmicutes, Bacteroidetes, Proteobacteria, and Actinobacteria. Additionally, these microorganisms participate in drug biotransformation and sustain immune homeostasis [82, 83]. Dysregulated gut microbiota and associated metabolites have been linked to multiple pathological conditions, including autoimmune disorders [84], gastrointestinal malignancies [85, 86], cardiovascular diseases [87], and neurodegenerative disorders [88]. Early investigations of the human microbiome primarily addressed its involvement in various glucose metabolism pathways, particularly glycolysis, whereas later research has revealed complex interactions between gut microbiota and host metabolic processes [89]. Gut microbiota dysbiosis frequently correlates with enhanced glycolytic activity in malignancies. A representative example is the elevated abundance of *Fusobacterium nucleatum* ( *F. nucleatum* ) in colorectal cancer patients, which demonstrates a strong association with dysregulated glycolysis activation in CRC pathogenesis [90]. Moreover, dysregulation of microbial metabolites frequently coexists with abnormalities in gut microbiota. Changes in gut microbiota and a reduction in metabolites of short‐chain fatty acids (SCFAs), which accelerate glycolysis, have been linked to an increased risk of colorectal cancer [91]. Crucially, circadian rhythmicity serves as a fundamental regulator of microbial homeostasis. Circadian disruption perturbs microbial composition and metabolic output, thereby reprogramming cellular metabolism [2]. Murine models with feeding rhythm disruption exhibit intestinal circadian desynchronization, marked microbial community alterations, and reduced butyrate (a key SCFA) levels that accelerate alcohol‐associated intestinal polyposis progression. Supplementation with butyrate‐producing prebiotics effectively attenuates tumor burden in these models [92]. Cancer metastasis correlates with adverse clinical outcomes across multiple malignancies. In CRC lung metastasis models, *Bmal1* or *Per1/2* deficiency induces gut microbiota remodeling with elevated taurocholic acid (TCA) production. This metabolite enhances glycolytic enzyme (ALDOA and ENO1) expression via H3K4 methylation in MDSCs, fostering immunosuppression and metastatic progression [1]. These researches illuminate complex circadian‐microbiota‐metabolite interactions in tumor metabolism, positioning microbial ecosystem stabilization as a promising therapeutic frontier.

## 4 Therapeutic Targeting of Glycolysis in Chronotherapeutic Strategies

Targeting tumor metabolic pathways, especially glycolysis, has gained significant attention as a therapeutic strategy in oncology [93]. Current research on glycolytic inhibitors focuses on suppressing glycolytic enzymes and their intermediates or directly inhibiting GLUTs and monocarboxylate transporters (MCTs), all leading to glycolysis blockade in cancer cells [94]. The metabolic plasticity of cancer cells enables adaptive utilization of diverse nutrients to sustain proliferation and survival. This adaptive capacity underscores the therapeutic advantage of combining metabolic inhibitors with conventional therapies over monotherapy [14]. The glucose analog 2‐deoxy‐D‐glucose (2‐DG), a noncompetitive hexokinase 2 inhibitor, manifests superior antitumor efficacy when combined with the chemotherapeutic agent sorafenib compared to either agent administered alone [95]. The novel GLUT1 inhibitor WZB117 exerts its anticancer activity through irreversible binding that potently blocks glucose transport. This inhibitor shows synergistic effects with cisplatin or paclitaxel in breast cancer models [96]. Emerging combination strategies integrating glycolytic inhibitors with targeted therapies or immunotherapies show enhanced antitumor potential. For instance, in gastric cancer, the glycolytic inhibitor dichloroacetate (DCA) potentiates anti‐PD‐L1 immune checkpoint blockade (ICB) efficacy by modulating pyruvate dehydrogenase kinase (PDK) activity [97].

Recent studies highlight the therapeutic potential of chrono‐targeting glycolytic pathways, capitalizing on the circadian regulation of glycolysis [98]. The glycolytic inhibitor PFK15 displays time‐dependent efficacy in mouse models of colorectal adenocarcinoma: morning administration significantly attenuates tumor growth through 6‐phosphofructo‐2‐kinase/fructose‐2,6‐bisphosphatase 3 (PFKFB3) inhibition, whereas equivalent evening dosing fails to replicate this antitumor effect [54]. Chronotherapeutic approaches achieve dual modulation by targeting circadian clock components to reset endogenous rhythms and optimizing drug administration timing for enhanced therapeutic outcomes [98]. Metformin is a hypoglycemic agent with glycolytic inhibitory properties and first‐line status in type 2 diabetes management [94]. It has been repurposed in oncology because of its established safety profile and patient tolerability [99]. In HER2‐amplified gastric cancer cells, disrupted *PER1* interacts with *PPARγ* to upregulate HK2 expression, thereby augmenting glycolytic flux and conferring trastuzumab resistance. Metformin counteracts this resistance through HK2 activity suppression. Interestingly, combined administration of metformin and trastuzumab during peak HK2 activity (ZT18) exhibits superior efficacy compared to trough‐phase treatment (ZT6), with preserved treatment tolerability [100]. These findings validate chronotherapeutic integration with targeted therapies as a viable treatment paradigm.

Accumulating clinical evidence shows circadian optimization of therapeutic interventions, with chemotherapy, radiotherapy, and immunotherapy exhibiting enhanced antitumor efficacy when administered during specific circadian phases [101, 102, 103]. Female patients with bone metastasis pain exhibit a superior response to noon‐administered radiotherapy compared to other time windows [104]. This underscores the need for systematic chronotherapeutic studies to optimize the administration timing of glycolytic inhibitors alongside conventional and emerging therapies for maximal therapeutic synergy.

## 5 Conclusions

Circadian disruption is critically implicated in tumorigenesis and malignant progression, particularly through glycolysis activation that fuels tumor proliferation. Circadian rhythm disruption drives tumor progression through multifaceted mechanisms of glycolytic activation. These processes involve related signaling molecules, genetic and transcriptional regulation, gut microbiota, and associated metabolites. Notably, the circadian regulation of glycolysis may vary depending on the tumor microenvironment and cell type. For instance, *BMAL1* knockdown enhances glycolysis in primary colon cancer cells but reduces it in metastatic cancer cell lines from mesenteric lymph nodes, whereas CAFs derived from CRC patients paradoxically demonstrate suppressed glycolytic activity [105]. These differences likely require further studies to identify the specific factors responsible. Current evidence indicates that circadian rhythm disruption enhances glycolytic flux across diverse tumor microenvironment components, including cancer cells, MDSCs, TAMs, and CAFs, thereby accelerating malignant progression. This review aims to delineate the mechanistic interplay between circadian dysregulation and glycolytic upregulation in tumor progression while elucidating the complex relationship connecting circadian biology, metabolic adaptation, and microenvironmental remodeling. These research advances our mechanistic understanding of tumor progression while providing a theoretical basis for innovative therapeutic development. The strategic combination of radiotherapy, chemotherapy, immunotherapy, targeted therapy, and glycolysis‐targeted therapies may improve clinical outcomes through chronotherapeutic optimization.

## 6 Recommendations and Future Perspectives

Current glycolytic inhibitors display measurable but limited antitumor efficacy, yet persistent challenges require systematic resolution through innovative therapeutic strategies. Glycolysis inhibitors have not been widely studied or applied clinically because of significant side effects [106]. This necessitates prioritizing the development of next‐generation medicines with improved selectivity and reduced toxicity. It also underscores the need to explore novel combination therapies with therapeutic potential. Studies show that activating GLP‐1 (glucagon‐like peptide 1) and the proteasome inhibits glycolysis, thereby suppressing tumors [107, 108]. On the basis of this, it is possible to repurpose drugs such as GLP‐1‐based therapies or proteasome‐targeted therapies, and use various natural compounds (e.g., prodigiosin or hinokitiol) as preventive medicines with immunomodulatory effects [109, 110], with a positive impact. Additionally, clinical trials indicate that optimized dosing of glycolysis inhibitors reduces drug‐related adverse events and enhances clinical benefits in combination therapies [111]. More importantly, chronotherapy can mitigate drug toxicity [112]. A metastatic colorectal cancer trial demonstrated that timed administration of triple chemotherapy reduced toxicity and improved first‐line drug efficacy [113]. These findings suggest that chronotherapy enhances antitumor efficacy while improving safety, thereby increasing the clinical potential of combination therapies. However, current clinical trials in this area remain limited, and more evidence is needed to determine whether chronotherapy‐optimized glycolysis inhibitor combinations can achieve clinical benefits and widespread adoption. Parallel research efforts should explore advanced technologies such as gene editing and nanotechnology to refine drug targeting and potency. Technological innovation coupled with interdisciplinary collaboration is expected to drive transformative progress in cancer therapeutics.

## Author Contributions

H.W. was responsible for manuscript drafting and visualization; D.W. and J.L. reviewed and critically edited the manuscript; B.Z. and L.L. participated in manuscript preparation; X.W. supervised the research and conceptualized the study. All authors read and approved the final manuscript. Data authentication is not applicable.

## Ethics Statement

The authors have nothing to report.

## Consent

The authors have nothing to report.

## Conflicts of Interest

The authors declare no conflicts of interest.

## Data Availability

The authors have nothing to report.
